# Molecular profiling of gastric cancer in a population with high HIV prevalence reveals a shift to MLH1 loss but not the EBV subtype

**DOI:** 10.1002/cam4.3001

**Published:** 2020-03-24

**Authors:** Violet Kayamba, Julia Butt, Tim Waterboer, Ellen Besa, Naheed Choudhry, Anglin Hamasuku, Peter Julius, Douglas C. Heimburger, Masharip Atadzhanov, Paul Kelly

**Affiliations:** ^1^ Tropical Gastroenterology & Nutrition Group Department of Internal Medicine Lusaka Zambia; ^2^ University of Zambia School of Medicine Lusaka Zambia; ^3^ Cancer Control and Population Sciences Program Duke Cancer Institute Duke University Durham NC USA; ^4^ Department of Population Health Sciences Duke University Durham NC USA; ^5^ Infection and Cancer Epidemiology Group German Cancer Research Center (DKFZ) Heidelberg Germany; ^6^ Blizard Institute Barts & The London School of Medicine and Dentistry Queen Mary University of London London UK; ^7^ University Teaching Hospital Lusaka Zambia; ^8^ Vanderbilt Institute for Global Health and Department of Medicine Vanderbilt University Medical Center Nashville TN USA

## Abstract

The human immunodeficiency virus (HIV) pandemic heavily affects sub‐Saharan Africa. It is associated with persistently active Epstein‐Barr virus (EBV) infection. To determine if this translates into increased frequency of EBV‐associated gastric cancer (EBVaGC), we evaluated molecular profiles of gastric cancer (GC) in Zambia. Patients with GC or premalignant gastric lesions were enrolled in Lusaka, Zambia. We used patients without any of these lesions as a control group. Chromogenic in situ hybridization (CISH) on tumor tissue was used to identify EBVaGC. To identify the microsatellite unstable subtype, immunofluorescence staining for MutL homolog 1 (MLH1) was used. Exposure to EBV and HIV was assessed serologically. We enrolled 369 patients (median age 52 years [IQR 41‐65]; 198 (54%) female). Of these, 72 (20%) had GC and 35 (9%) had gastric premalignant lesions (PL). CISH identified EBVaGC in 5/44 (11%) of those with adequate tissue, while MLH1 loss was identified in 29/45 (64%). Both GC and PL were associated with the highest titers of antibodies to Early antigen‐diffuse (OR 2.5, 95% CI 1.0‐6.1, *P* = .048 and OR 3.9, 95% CI 1.1‐12.9, *P* = .03, respectively) at high concentrations. Human immunodeficiency virus infection was associated with a range of antibodies to EBV, but not with any cancer subtype. Despite the association of HIV with persistent EBV, the proportion of EBVaGC in Zambia is similar to populations with a low prevalence of HIV infection. The proportion of microsatellite unstable tumors may be higher than other populations.

## Graphical abstract

Human immunodeficiency virus infection is associated active Epstein‐Barr virus (EBV) infection. We found that this does not necessarily translate into an increased proportion of EBV‐associated gastric cancer. We also found a much higher proportion of microsatellite unstable tumors than that reported elsewhere. These findings provide good preliminary data that could be helpful in understanding not only gastric carcinogenesis but probably avenues for new therapies as well.

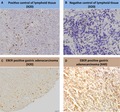

## 1 INTRODUCTION

Gastric cancer (GC) is one of the leading causes of cancer‐related deaths in the world.^1^ It has very poor patient outcomes mainly due to late diagnosis and limited therapeutic options.^2^, ^3^, ^4^ Sub‐Saharan Africa has the highest burden of human immunodeficiency virus (HIV), with Zambia's adult population prevalence being 11.3%.^5^ While it has no known link to GC,^6^ HIV infection has been shown to influence the persistence of active Epstein‐Barr virus (EBV) infection.^7^, ^8^

Epstein‐Barr virus is a DNA virus identified as a class one carcinogen.^9^ It is known that 90% of the global population has evidence of prior exposure or active infection.^10^, ^11^ Epstein‐Barr virus has been implicated in the development of about 10% of GCs.^12^, ^13^, ^14^ Epstein‐Barr virus‐associated GC (EBVaGC) was one of the subtypes described by The Cancer Genome Atlas (TCGA) published in 2014.^12^ To define the molecular subtypes of GC, TCGA used several techniques including mRNA expression, exome sequencing, copy number Analysis, microRNA expression (miRNA‐seq), DNA methylation (450K arrays), protein phosphorylation analysis, and low‐pass whole‐genome sequencing on a subset. These analyses demonstrated that EBVaGC exhibits a high prevalence of DNA hypermethylation and a strong predilection for mutations in the phosphatidylinositol 3‐kinase, catalytic subunit alpha gene. This has potential as a therapeutic target for EBVaGC.^15^ Clinically EBVaGC has a relatively low rate of lymph node metastasis conferring a slightly better prognosis with a lower number of differentially expressed genes.^16^, ^17^ In addition, EBVaGC is more common in younger patients.^18^

Three other GC subtypes described by TCGA were genomically stable, high chromosomal instability, and microsatellite unstable tumors. Microsatellite unstable tumors tend to display loss of MutL homolog 1 (MLH1) expression which can be demonstrated using immunostaining.^19^ There is no information on the occurrence of microsatellite unstable GC in high HIV prevalent populations such as Zambia.

With the knowledge that HIV‐infected individuals were more likely to have active EBV infection than uninfected,^8^, ^20^ we hypothesized that in Zambia, the prevalence of EBVaGC would be higher than that reported from areas with a low burden of HIV infection. It is also known that persistent HIV viremia and immune activation favors the onset of EBV‐related malignancies, and many malignant diseases that arise in the setting of HIV infection tend to present at a more advanced stage with shorter survival time.^21^, ^22^, ^23^

We, in addition, used multiplex serology to quantify specific EBV antibodies against Early antigen (EA‐D), viral capsid antigen (VCA p18), Epstein‐Barr nuclear antigen (EBNA), and BZLF1‐encoded replication activator protein (ZEBRA). Epstein‐Barr nuclear antigen is a sequence‐specific DNA binding phosphoprotein with a central role in maintaining latent EBV infection. It is required for the replication and maintenance of the EBV genome.^24^ BZLF1‐encoded replication activator protein is a transcription factor involved in conversion of the virus from the latent to lytic phase.^25^ In primary infection antibodies are produced against EA‐D and their persistence signifies active infection.^26^ These antibodies were compared between patients with GC and those without and patients with HIV infection and those without. To our knowledge, the influence of HIV infection on the development of EBVaGC has not been reported.

## 2 METHODS

This was a cross‐sectional study, and the analysis was conducted at two levels, first with gastric adenocarcinoma and second with premalignant lesions (PL) (chronic atrophic gastritis or gastric intestinal metaplasia). The study was conducted at the Endoscopy Unit of the University Teaching Hopsital located in Lusaka, the capital city of Zambia with patients referred for specialist care from all 10 provinces of Zambia. All patients referred for diagnostic oesophagogastroduodenoscopy between July 2016 and April 2018 were considered for enrollment. The inclusion criteria were patients above the age above 18 years, having given full written consent to participate. Excluded were those with prior history of treatment for GC, history of ingesting a caustic substance, or unwillingness to have an HIV test.

### 2.1 Oesophagogastroduodenoscopy and study questionnaire

All patients came to the study unit having starved overnight. A complete evaluation of the mucosa was carried out and six biopsies taken from any gastric lesions. In addition, if no lesion suspicious of being GC was seen, two biopsies each were taken from the antrum, incisura, and body. Biopsies were then fixed in 10% neutral buffered formalin for histopathological analysis. After the procedure, 10 mL of blood was collected from each participant for serum extraction, which was then stored at −80°C. Interviews were conducted using a structured questionnaire to collect demographic characteristics.

### 2.2 Histopathology and case ascertainment

Patients with chronic atrophic gastritis or gastric intestinal metaplasia were grouped together as having PL. Gastric adenocarcinomas were divided using the Lauren classification into Intestinal type and diffuse.

### 2.3 Epstein Barr virus in gastric tumors

**FIGURE 1:**
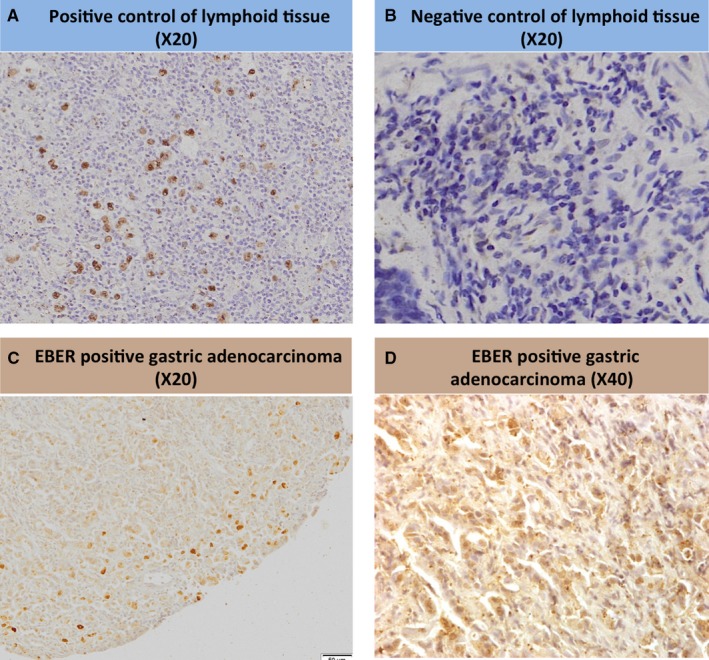
Epstein‐Barr encoding region (EBER) in situ hybridisation images; (A) slide used as a positive control; (B) slide used as a negative control; (C) positive slide showing dark spots of EBER in gastric adenocarcinoma and (D) slide (C) at a higher power

EBV‐encoded RNA (EBER) chromogenic in situ hybridization (CISH) technique was used on formalin‐fixed paraffin‐embedded gastric tumor biopsies (EBV CISH Detection Kit; Master Diagnostica) following the manufacturer's instructions. In situ hybridization allows for the detection of EBV DNA without obscuring cytopathological detail. Digoxigenin‐labeled 5′ RNA oligonucleotides complementary to type 1 and 2 EBER of EBV were used for the detection of the presence of EBV‐infected cells in latency (Figure 1).

### 2.4 Immunofluorescence staining for MLH1 in GC

**FIGURE 2:**
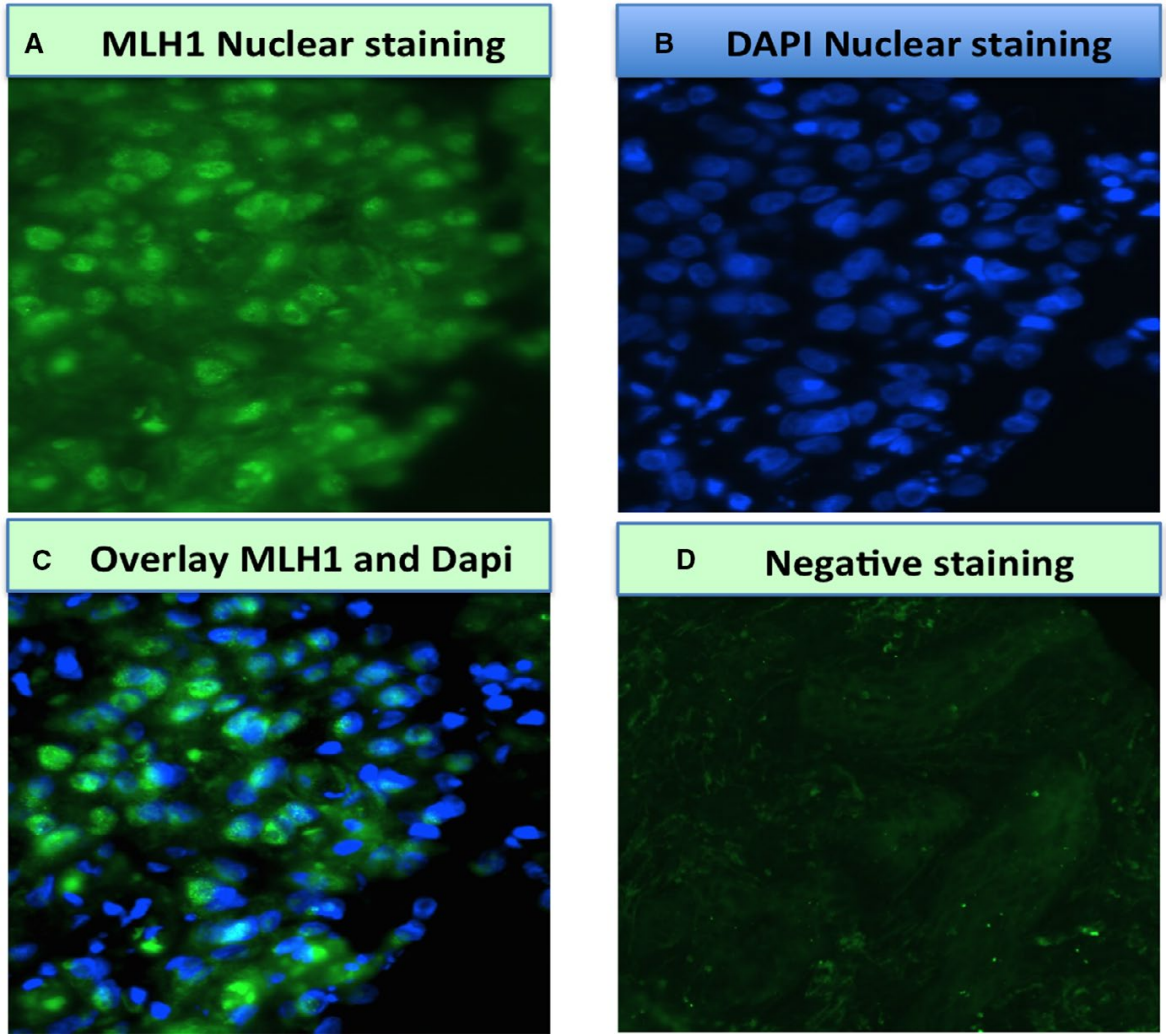
Immunofluorescence staining for MutL homolog 1 (MLH1); (A) positive staining for MLH1, (B) 4,6‐diamidino‐2‐phenylindole (DAPI) nuclear staining, (C) overlay of (A) and (B), (D) negative control

Formalin‐fixed paraffin‐embedded biopsies were sectioned and mounted onto poly‐lysine slides. These were then placed in a 60°C incubator overnight, after which they were deparaffinized and hydrated in xylene and ethanol, respectively. Antigen retrieval was done using a preheated citrate‐based buffer (Vector Laboratories) for 20 minutes. The tissue was then washed in phosphate buffered saline (PBS) before being permeabilized using 0.2% Tween (Sigma‐Aldrich) in PBS. Blocking was done using 20% goat serum in PBS to prevent nonspecific binding, and then incubated with primary antibody overnight (anti‐MLH1 antibody ab92312; Abcam) at a dilution of 1:500. The tissue was then washed again in PBS before incubation with the conjugated secondary antibody (goat anti‐rabbit ab150077; Abcam) at a 1:50 dilution for 1 hour in the dark. Once the hour had elapsed, the tissue was washed a third time, and then mounted onto the slide using a mounting medium with 4,6‐diamidino‐2‐phenylindole (VECTASHIELD; Vector laboratories). For each run, positive and negative controls were included. Examples of positive and negative MLH1 immunofluorescence staining are shown in Figure 2.

### 2.5 Multiplex assay to test for EBV

Antibody responses to EBV antigens EA‐D, VCA p18, EBNA, and ZEBRA were performed using multiplex serology as previously described.^27^ Briefly, EBV antigens were recombinantly expressed as glutathione‐S‐transferase‐tagged proteins in *Escherichia coli*. Proteins were affinity purified on glutathione‐coupled fluorescent beads (Luminex Corp.), each antigen on a bead type with a distinct internal fluorescence. Antigen‐coupled beads were mixed and incubated with serum (dilution 1:1000). Bound serum antibodies were quantified using a biotin‐coupled secondary anti‐immunoglobulin‐IgA/IgM/IgG antibody and a streptavidin‐coupled reporter fluorescence (phycoerythrin). A Luminex analyzer (Luminex Corp.) then distinguished between the bead type and consequently the bound antigen as well as quantified the amount of bound serum antibody by determining the median fluorescence intensity (MFI) on at least 100 beads per type measured. The cut‐offs for antigen‐specific seropositivity were as follows: EA‐D 300 MFI, VCA p18 2500 MFI, EBNA 1800 MFI, and Zebra 200 MFI.

### 2.6 Testing for HIV

Serum samples were tested for the presence of HIV antibodies using Uni‐Gold™ rapid diagnostic kits (Trinity Biotech).

### 2.7 Data analysis and sample size calculation

To summarize continuous variables, medians and interquartile ranges were used. Associations among GC, PL, and HIV and EBV infection were sought using Fisher's exact test, and expressed using odds ratio and 95% confidence intervals. Unconditional logistic regression was employed to assess the relative contributions of the antibody titers divided into quartiles and using the seronegative results as the reference. The Kruskal‐Wallis test was used for hypothesis testing of continuous variables. In all cases, a two‐sided *P* < .05 was considered statistically significant. To calculate the sample size, we used estimates from a previous study in Zambia in which we found that 29% of GC patients and 9% of the controls had positive antibodies to EBV EA.^8^ With a ratio of one case to three controls, we needed at least 49 cases and 147 controls to be able to reject the null hypothesis that this odds ratio equals 1 with probability (power) 0.8. The type 1 error probability associated with this test of the null hypothesis was 0.05. The statistical package used was STATA version 15 (College Station, TX, USA). All data used in this manuscript can be made available upon request.

## 3 RESULTS

### 3.1 Enrolment of study participants and histological classification

We enrolled 369 patients, of which 68 (18%) had histologically confirmed gastric adenocarcinoma here referred to as GC. In addition, four patients with obvious gastric tumor masses and histological evidence of high‐grade dysplasia (carcinoma in situ) were included as cases, bringing the total number of cases to 72 (20%). Premalignant lesions were present in 35 (9%) patients including chronic atrophic gastritis without intestinal metaplasia (n = 8) and intestinal metaplasia (n = 27). The remaining 262 (71%) had nonatrophic gastritis and were included as the control group. Of the GC seen during endoscopy, 25 (35%) were proximal while 47 (65%) were distal tumors. Forty‐five tumors were classified using the Lauren classification with 15 (34%) being diffuse, 28 (62%) intestinal, and 2 (4%) being of mixed subtype.

### 3.2 Basic characteristics of patients with GC, PL, and controls

The distribution of GC cases was similar between males and females, but GC cases and those with PL were significantly older than the controls (Table 1). Age difference was therefore corrected for when analyzing for associations. Gastric cancer was associated with rural residence and a low body mass index. Reported alcohol intake and cigarette smoking were similar between cases and controls. The prevalence of HIV infection was 21%; it was not associated with GC or PL (Table 1).

**TABLE 1:** Basic characteristics of patients with gastric cancer or premalignant lesions and those without these lesions

	Controls n = 244	Gastric cancer n = 72	OR; 95% CI	*P* value	Premalignant lesions n = 35	OR; 95% CI	*P* value
Gender							
Female	128 (52%)	39 (54%)	1.1; 0.5‐1.9	.89	17 (49%)	0.9; 0.4‐1.9	.72
Male	116 (48%)	33 (46%)			18 (51%)		
Age							
Less than 30 y	10 (4%)	1 (2%)			0 (0%)		
30‐44 y	89 (37%)	10 (14%)			10 (29%)		
45‐59 y	86 (35%)	24 (33%)			6 (17%)		
60 y and above	59 (24%)	37 (51%)	—	**.0001**	19 (54%)	—	**.005**
Resident in rural area	45 (18%)	28 (39%)	2.9; 1.5‐5.3	**.0004**	8 (23%)	1.3; 0.5‐3.2	.50
Body mass index, median (IQR)	25 (21‐28)	18 (16‐21)	—	**.0001**	26 (23‐28)	—	.37
Married	151 (62%)	40 (56%)	0.8; 0.4‐1.4	.41	24 (71%)	1.5; 0.6‐3.6	.45
Educational level attained							
None	16 (7%)	13 (18%)			1 (3%)		
Primary	57 (23%)	27 (37%)			13 (37%)		
Secondary	98 (40%)	20 (28%)			15 (43%)		
Tertiary	74 (30%)	12 (17%)	—	**.0001**	6 (17%)	—	.13
No employment	58 (24%)	26 (36%)	1.8; 1.0‐3.3	**.048**	15 (43%)	2.4; 1.1‐5.3	**.02**
Family history of gastric cancer	6 (2%)	1 (1%)	0.6; 0.01‐4.7	1.00	1 (3%)	1.2; 0.02‐10.1	.39
History of smoking							
Current	12 (5%)	7 (10%)	2.3; 0.7‐6.7	.21	3 (9%)	2.1; 0.3‐8.3	.39
Ever	23 (9%)	12 (17%)	2.0; 0.9‐4.6	.08	3 (9%)	1.0; 0.2‐3.4	1.00
History of alcohol intake	58 (24%)	12 (17%)	0.6; 0.3‐1.3	.21	6 (17%)	0.7; 0.2‐1.8	.51

Values in bold were statistically significant.

### 3.3 Molecular profiling of GC

In order to determine the proportion of cases attributable to EBV and microsatellite instability, we checked for EBV and MLH1. Using CISH for EBER, 44 slides yielded successful results for interpretation. Forty‐five slides were successfully stained for MLH1. The remaining paraffin embedded blocks did not have enough identifiable tumor tissue for successful staining or interpretation. EBER was detected in 5/44 (11%) cases. EBER positivity was not associated with the type of tumor (by Lauren classification; *P* = .31), anatomical location within the stomach (*P* = 1.00), age (*P* = .54), or gender (*P* = .22). Of the gastric biopsies successfully stained for MLH1, 15/45 (36%) showed positive nuclear staining, and therefore 29/45 (64%) had evidence of microsatellite instability. Seventy‐four percent of GC patients whose age was 50 and above years showed microsatellite instability as opposed to 30% among those below that age (OR 6.7; 95% CI 1.2‐47, *P* = .02). There was, however, no association with Lauren classification (*P* = .93), anatomical location within the stomach (*P* = .38), or gender (*P* = .35). There was, therefore, no evidence of an increase in EBV‐related GC, rather an increase in microsatellite unstable tumors compared with the original TCGA description.

### 3.4 Association between GC OR PL and EBV serology

In order to assess EBV exposure, we analyzed four EBV antibodies in serum, including EA‐D, VCA p18, EBNA, and ZEBRA. These were compared between patients with GC or PL and controls. EBV serology antibody results were available for 59 GC, 27 PL, and 210 controls. Overall, 98% of the patients had one or more EBV antibodies in their serum. The presence of EBV antibodies was similar between GC cases and controls (Table 2). Similarly, there was no association between PL and VCA p18, EBNA, or ZEBRA. However, having PL was associated with EA‐D positivity, OR 2.5, 95% CI 1.0‐7.4, *P* = .04, (Table 2). We then analyzed antibody MFI values by dividing them into quartiles, using negative readings as a reference and controlling for age and gender. Both GC and PL were associated with the highest EA‐D levels of the 4th quartile but not the lower quartiles. There was no association observed with EBNA, VCA p18, or ZEBRA (Table 3).

**TABLE 2:** Association of gastric cancer, premalignant lesions, and HIV with the presence of EBV antigens

Antibody (cut‐off)	Controls	Gastric cancer	OR; 95% CI	*P* value	Pre malignant lesions n = 27	OR; 95% CI	*P* value	HIV positive n = 56	HIV negative n = 232	OR; 95% CI	*P* value
Early antigen (300)	111(53%)	3 (66%)	1.7; 0.9‐3.4	.08	20 (74%)	2.5; 1.0‐7.4	.04	45 (80%)	128 (53%)	3.7; 1.8‐8.2	.0001
Viral capsid antigen (2500)	188 (90%)	55 (93%)	1.6; 0.5‐6.7	.47	16 (96%)	3.0; 0.4‐130	.49	56 (100%)	216 (89%)	—	.004
Epstein‐Barr nuclear antigen (1800)	105 (98%)	59 (100%)	—	.59	26 (96%)	0.6; 0.1‐31	.52	52 (93%)	240 (99%)	0.2; 0.02‐1	.03
BZLF1‐encoded replication activator protein (200)	169 (80%)	47 (80%)	1.0; 0.4‐2.1	.86	26 (96%)	6.3; 1.0‐264	.06	54 (96%)	193 (79%)	7.0; 1.7‐60	.001

Abbreviations: EBV, Epstein‐Barr virus; HIV, human immunodeficiency virus.

**TABLE 3:** Quantitative analysis of EBV antibodies compared between cases (gastric cancer or premalignant lesions) against controls

	Controls n = 210	Gastric cancer n = 59	OR; 95% CI	*P* value	Premalignant lesions n = 27	OR; 95% CI	*P* value	HIV positive n = 56	HIV negative n = 243	OR; 95% CI	*P* value
	n (%)	n (%)			n (%)						
Early antigen (300)											
Negative	99 (47)	20 (34)	1.00 (ref)		7 (26)	1.00 (ref)		11 (20)	111(48)	1.00 (ref)	
MFI 1st quartile	27 (13)	11 (19)	1.5; 0.6‐3.8	.384	5 (18)	3.0; 0.8‐11	.102	3 (5)	39 (17)	1.0; 0.2‐3.9	.981
MFI 2nd quartile	30 (14)	9 (15)	1.3; 0.5‐3.2	.615	4 (15)	1.6; 0.4‐6.1	.502	6 (11)	34 (14)	2.8; 0.9‐8.7	.074
MFI 3rd quartile	32 (15)	7 (12)	0.9; 0.3‐2.4	.817	4 (15)	1.6; 0.4‐6.3	.480	12 (22)	30 (13)	6.4; 2.3‐17.2	**<.001**
MFI 4th quartile	22 (11)	12 (20)	2.5; 1.0‐6.1	**.048**	7 (26)	3.9; 1.1‐12.9	**.027**	23 (42)	18 (8)	26.7; 9.5‐74.8	**<.001**
Viral capsid antigen (2500)											
Negative	22 (11)	4 (7)	1.00 (ref)		1 (4)	1.00 (ref)		0 (0)	26 (11)	1.00 (ref)	
MFI 1st quartile	44 (21)	14 (24)	2.7; 0.7‐10	.142	10 (37)	6.1; 0.7‐54	.105	4 (7)	62 (27)	0.1; 0.02‐0.3	**<.001**
MFI 2nd quartile	51 (24)	11 (19)	1.5; 0.4‐5.9	.523	7 (26)	3.8; 0.4‐31	.284	11 (20)	55 (24)	0.3; 0.1‐0.8	**.010**
MFI 3rd quartile	47 (22)	15 (25)	2.5; 0.7‐9.2	.171	2 (7)	1.7; 0.1‐16	.836	17 (31)	44 (19)	0.6; 0.3‐1.4	.270
MFI 4th quartile	46 (22)	15 (25)	2.1; 0.6‐7.8	.254	7 (26)	4.2; 0.4‐34	.249	23 (42)	45 (19)	—	—
Epstein‐Barr nuclear antigen (1800)											
Negative	5 (2)	0 (0)	1.00 (ref)		1 (4)	1.00 (ref)		3 (5)	3 (1)	1.00 (ref)	
MFI 1st quartile	48 (23)	18 (30)	1.9; 0.8‐4.4	.141	6 (22)	0.7; 0.1‐8.7	.790	19 (35)	51 (22)	0.3; 0.1‐1.8	.198
MFI 2nd quartile	52 (25)	14 (24)	1.1; 0.5‐2.7	.793	6 (22)	0.7; 0.1‐9.1	.815	11 (20)	59 (26)	0.2; 0.02‐1.0	.050
MFI 3rd quartile	46 (22)	13 (22)	1.3; 0.5‐3.2	.568	8 (30)	1.0; 0.1‐12.4	.970	12 (22)	52 (22)	0.2; 0.03‐1.2	.076
MFI 4th quartile	59 (28)	14 (24)	—	—	6 (22)	0.6; 0.05‐7.3	.687	10 (18)	67 (29)	0.1; 0.02‐0.7	**.023**
BZLF1‐encoded replication activator protein (200)											
Negative	41 (20)	12 (20)	1.00 (ref)		1 (4)	1.00 (ref)		2 (4)	49 (21)	1.00 (ref)	
MFI 1st quartile	44 (21)	10 (17)	0.7; 0.2‐1.8	.406	8 (29)	6.3; 0.7‐54.9	.094	7 (13)	55 (24)	4.3; 0.8‐22.7	.086
MFI 2nd quartile	47 (22)	8 (14)	0.6; 0.2‐1.8	.396	7 (26)	7.7; 0.9‐68	.065	7 (13)	52 (23)	3.9; 0.7‐20.6	.107
MFI 3rd quartile	42 (20)	14 (24)	1.0; 0.4‐2.6	.993	4 (15)	3.5; 0.4‐34.2	.276	13 (23)	45 (19)	9.6; 1.9‐47.6	**.005**
MFI 4th quartile	36 (17)	15 (25)	1.0; 0.4‐2.6	.962	7 (26)	5.8; 0.7‐50.9	.114	26 (47)	31 (13)	50.8; 9.9‐260	**<.001**

Abbreviations: EBV, Epstein‐Barr virus; HIV, human immunodeficiency virus; MFI, median fluorescence intensity.

Values in bold were statistically significant

### 3.5 Association between HIV infection and EBV serology

To assess how HIV infection impacts on EBV exposure, we compared EBV antibodies in HIV‐positive (n = 56) and HIV‐negative (n = 243) patients irrespective of their clinical or histopathological diagnosis. There was an association between HIV infection and having antibodies to EA‐D, VCA p18, and ZEBRA (Table 2), but not antibodies to EBNA which were almost ubiquitous. When the MFI values were subdivided into quartiles as above, being HIV positive was associated with higher values for EA‐D, EBNA, and ZEBRA but lower values for VCA p18 (Table 3). Therefore, HIV infection was associated with increased serological evidence of EBV infection.

## 4 DISCUSSION

As HIV infection is associated with persistent EBV activity,^8^ an association we confirmed in our study, we sought to determine if this translates into increased frequency of EBVaGC, possibly explaining the frequent occurrence of early‐onset cancers. We found no evidence for this: the proportion of EBVaGC was similar to that reported from regions where the HIV burden is low and EBV exposure lower. Instead, we found a considerably higher proportion of microsatellite unstable GC than has been reported elsewhere.^12^, ^28^

Epstein‐Barr virus is transmitted from host to host via saliva and over 90% of the world's population is infected.^10^, ^29^ Mechanisms involved in EBV‐associated gastric carcinogenesis are a subject of continued investigation. One suggestion is that EBV induces a delay in apoptosis and cellular differentiation. Our findings suggest that HIV does not influence gastric carcinogenesis despite promoting continued EBV activity. There are various techniques that can be employed to identify EBVaGC. We used in situ hybridization, which is considered the gold standard for the presence of replicating EBV in a histopathological lesion.^30^ This technique demonstrates EBV‐encoded RNA (EBER), which is nonpolyadenylated, uncapped noncoding RNA, in gastric tumor cells.^31^

EBER is expressed in almost all EBV‐infected cells. The proportion of GC attributable to EBV was almost exactly the same as in original TCGA report from western and Asian countries.

By contrast, we found a much higher proportion of microsatellite unstable GC than the 13%‐44% reported in the literature.^12^, ^28^ Microsatellite unstable tumors are characterized by hypermutation which could be induced by environmental factors. The higher proportion of MLH1 loss found in this study, therefore, warrants further investigation for its relationship with environmental risk factors. Loss of MLH1 was higher among patients above the age of 50 years and less so in early onset cancers. Similarly, Carvalho et al^32^ reported that patients with early onset gastric adenocarcinoma all had MLH1‐positive tumors without germline mutations of CDH1, TP53, or RUNX3.

As the presence of EBV antibodies showed no significant association with GC, we performed a quantitative analysis of these antibodies foregoing any predetermined cut‐offs. There was an association between GC and EA‐D but not ZEBRA, VCA p18, or EBNA. These findings are similar to our previous work, in which we found that 29% of GC patients were EA‐D–positive against only 9% of the controls.^8^ EA‐D antibodies are produced during the early phase of EBV replication. Their presence signifies a new infection or viral re‐activation. The association of GC and this antibody suggests that viral re‐activation is either a cause of or a result of EBVaGC. Antibodies to EBNA remain positive during the convalescent stages of EBV infection and it is therefore not surprising that we found no statistically significant association with GC. Similarly, VCA p18 despite being positive in early infection, it also remains detectable during convalescent stages of the infection.

In this study, HIV infection was associated with markers of primary EBV infection (EA‐D, VCA p18, and ZEBRA), but not the marker of chronic infection EBNA. Quantitative analysis of the EBV antibodies showed that differences between HIV‐infected and ‐uninfected individuals were most pronounced at higher antibody concentrations. As EBV is largely acquired in early in life, the high occurrence of markers of primary infection could be signifying viral re‐activation following HIV infection. It is thought that immunodeficient states, such as in HIV, permit spontaneous replication of the episomal virus in circulating B cells.^23^ In contrast, immunocompetent individuals control latent infection. We previously showed that GC was not associated with HIV infection and that antiretroviral therapy did not significantly alter measures of gastric physiology.^6^, ^33^ This study has similarly shown no association between GC and HIV infection.

We are concerned that the modest sample size might have led to an underestimate of the proportion of EBVaGC. However, the proportion we observed is consistent with the global literature and our power calculation suggests that this sample size was probably adequate for our purpose. We were able to detect an increased proportion of microsatellite unstable GC. Serological evaluation of EBV antibodies has the limitation that the infection may be harbored in any one of myriad sites within the body. Therefore, the serological associations with gastric pathology should be interpreted with caution.

## 5 CONCLUSIONS

From these findings, we draw the conclusion that despite the high seroprevalence of HIV in Zambian adults, and despite the increased serological evidence of EBV activity in HIV‐infected adults, the proportion of EBVaGC in Zambia was similar to populations with a low prevalence of HIV infection. Therefore, HIV infection does not influence the occurrence of EBVaGC. The proportion of microsatellite unstable tumors is higher than reports from other populations. As microsatellite instability was mostly found in older patients, this observation does not explain the previously reported high prevalence of early onset GC in Zambia.

## CONFLICT OF INTEREST

The authors declare that they have no competing interests.

## AUTHOR CONTRIBUTIONS

VK, DCH, MA, and PK conceptualized the study. Patient enrollment was done by VK Specimen preparation and laboratory assays were done by VK, JB, TW, EB, NC, AH, and PJ All the authors contributed to the manuscript and approved the final version.

## ETHICS APPROVAL AND CONSENT TO PARTICIPATE

The University of Zambia Biomedical Research Ethics Committee (reference number 005‐03‐16) and the National Health Research Authority granted ethical approval for this study**.**

## CONSENT FOR PUBLICATION

All study patients gave well‐informed written consent to participate in the study.

## Data Availability

The datasets used and/or analyzed during this study are available from the corresponding author on reasonable request.
